# Severe Sunburns and Sunbed Use Risk with Cutaneous Melanoma: A Case–Control Study in Lithuania †

**DOI:** 10.3390/medicina61111941

**Published:** 2025-10-29

**Authors:** Grinvydas Butrimas, Lukas Šemeklis, Renata Paukštaitienė, Augustė Dubinskaitė, Ugnė Janonytė, Dalia Lukšienė, Skaidra Valiukevičienė

**Affiliations:** 1 Department of Skin and Venereal Diseases, Medical Academy, Lithuanian University of Health Sciences (LSMU), LT-50161 Kaunas, Lithuania; 2 Department of Ophthalmology, Medical Academy, Lithuanian University of Health Sciences (LSMU), LT-50161 Kaunas, Lithuania; 3 Department of Physics, Mathematics and Biophysics, Medical Academy, Lithuanian University of Health Sciences (LSMU), LT-44307 Kaunas, Lithuania; 4 Laboratory of Population Studies, Institute of Cardiology, Lithuanian University of Health Sciences (LSMU), LT-50103 Kaunas, Lithuania

**Keywords:** sun exposure, ultraviolet radiation, sunbeds, tanning, melanocytic nevi, cutaneous melanoma

## Abstract

*Background and Objectives*: To our knowledge, this is the first case–control study conducted in the Baltic countries that identified CM risk factors focusing on the investigated subjects’ phenotypic traits, severe sunburns, sunscreen and tanning bed use. *Materials and Methods*: This study analyses 180 subjects with CM (case group) and 182 randomly selected controls. All participants completed surveys about sun exposure habits and skin phototype (I–IV) according to the Fitzpatrick scale. Skin, eye, and hair colour, number of freckles, and melanocytic nevi greater than 2 mm in diameter (MN > 2 mm) on sites with maximal, intermittent, and minimal sun exposure were examined. *Results*: We determined five risk factors that significantly increased the odds ratio of CM (OR; 95% confidence interval): tanning bed use compared to non-use (6.46; 1.89–22.96), lack of sunscreen use compared to regular use (7.41; 2.88–19.09), and fair skin compared to medium and olive skin (2.06; 1.03–4.09). The probability of CM also increased with each instance of severe sunburn (2.57; 1.96–3.38) and with each additional MN > 2 mm in sun-exposed areas (1.05; 1.03–1.07). *Conclusions*: The results of this case–control study offer novel insights into modifiable risk factors for CM, highlighting potential targets for primary prevention strategies in the Baltic countries’ population, with dominant fair phenotypic traits of skin, eye, and hair colour.

## 1. Introduction

Cutaneous melanoma (CM) is a highly aggressive skin cancer primarily originating from melanocytes [1]. A global trend analysis has revealed an increase in CM incidence across Europe from 1990 to 2019, with Eastern Europe (including the Baltic states) exhibiting the greatest increase of 3.21% [2]. Analysis of epidemiological databases of CM from Latvia (2007–2017), Estonia (2007–2015), and Lithuania (2007–2013) shows a rising incidence of CM cases in all three Baltic countries: from 6.9 to 11.8 per 100,000 in Latvia, from 11.2 to 20.7 in Estonia, and from 8.2 to 10.5 in Lithuania [3]. Among Northern European countries, the Baltic states show relatively low CM incidence, but comparatively high mortality. Data from the European Cancer Information System (ECIS, 2017) reported CM incidence rates of 10.6, 12.4, and 17.1 per 100,000 in Latvia, Lithuania, and Estonia, respectively, with similar mortality rates of 3.5–3.9 per 100,000 [4]. However, in Norway, the incidence reached 46.6 per 100,000 and mortality 6.3 per 100,000 [4], which may suggest a higher detection rate and more effective early treatment compared to the Baltic states. This trend encourages the search for new measures for early diagnosis and prevention of CM.

Over the past decade, case–control and prospective cohort studies in adults have identified that Fitzpatrick skin phototypes I–II, light hair/eyes [5,6,7,8,9,10], the number of melanocytic nevi (MN) [9,10,11,12,13], and the presence of atypical MN (AMN) [9,11,14] are associated with an increased risk of CM. Ultraviolet (UV) radiation is also a well-established CM risk factor. Intermittent UV exposure, such as instances of sunburns [9,15,16,17,18,19], tanning [7,9,14,20], is known to have a significant relationship with the risk of CM. Continuous occupational UV exposure [21] and lifetime ambient UV levels [22] are also noted as risk factors for CM. Nonetheless, some studies have contradictory results and show no significant association with CM and occupational UV exposure [23,24], cumulative ambient UV radiation in adulthood [19,25,26] or childhood [26], and tanning [19]. It is important to note that the mentioned studies were performed in countries with different UV exposure and phenotypic traits of the investigated subjects. Thus, comparing these studies can be challenging, as populations with similar ancestry but contrasting levels of sun exposure may exhibit different CM risk [27], and the evaluation of regionally relevant risk factors plays a crucial role in the prevention of CM.

Cross-sectional studies conducted with Lithuanian children show that sunburns, light skin colour (I–II skin phototype), and extensive facial freckling are associated with a higher density of MN [28,29], which are known to be associated with CM risk. Moreover, the results of our previous case–control studies found that, in the Lithuanian population, several factors were linked to increased CM risk: a higher number of MN on the face and outer arms and hands (2–5 mm and ≥5 mm in diameter), the presence of iris freckles, a history of sunburn, and fair skin type [30,31].

To our knowledge, no research conducted in the Baltic countries has investigated the relationship between UV-related factors and CM risk. Our study provides information on sun exposure-related factors, including tanning bed use, sunscreen habits, sunburn frequency, and their associations with CM risk. Genetic studies have demonstrated that Lithuanians, Latvians, and—to a large extent—Estonians belong to the same gene pool cluster and share ancestral alleles, possibly including pigmentation-associated variants, suggesting similar phenotypic traits relevant to CM development [32,33]. Therefore, this case–control study offers novel insights into modifiable risk factors for CM, highlighting potential targets for prevention strategies in the Baltic countries with dominant fair phenotypic traits of light skin, hair, and eye colour.

## 2. Materials and Methods

### 2.1. Study Design

This case–control study analysed 180 subjects with CM, sourced from the Hospital of the Lithuanian University of Health Sciences Kauno klinikos (LSMUL KK) database. The case study group consisted of patients who were diagnosed with stage I–IV CM at the Department of Skin and Venerological Diseases of LSMUL KK from 2017 to 2023, according to the American Cancer Society classification of CM stages. In 2017–2023, 419 cases of CM were identified in the multidisciplinary council of the Skin and Venerological Diseases Clinic of the LSMUL KK. The invitation of the subjects to the study took place during the visits of these patients to the dermatovenerologist for CM monitoring. In total, 69.1% of the invited patients with CM agreed to participate in the study. The study was carried out following the Helsinki Declaration principles [34] and approved by the Kaunas Regional Biomedical Research Ethics Committee (9 June 2021 No. BE-2-66).

### 2.2. Participants

This study included 362 subjects. The average age of the subjects was 58.56 years. The case group consisted of 180 subjects, and the control group consisted of 182 subjects. In the control group, women made up 62.1% of subjects, and in the case group, 62.8% of subjects. The higher inclusion of women than men in the study was consistent with the health statistics data provided by the Institute of Hygiene, according to which women made up 63.9% of patients diagnosed with CM [35]. The groups of subjects did not differ significantly in terms of age, height, or weight. The control group consisted of 182 individuals with no history of dermatological disorders, who were selected from all responders (N = 3426) in the epidemiological health survey study “Chronic diseases and their risk factors in the adult population”, which was performed in Kaunas city (Lithuania). Criteria for inclusion of subjects in the control group of the study: Caucasians over 18 years of age, willing to participate in the biomedical study, and not diagnosed with CM during dermatological examination. Exclusion criteria, applied to both case and control groups, included severe systemic diseases, mental disorders affecting comprehension or participation, and pregnancy or lactation. Individuals unwilling or withdrawing consent during the study were also excluded.

### 2.3. Data Collection

All participants underwent dermatological examination. For each subject, eye colour (blue, grey, green, or brown), hair colour (red, blonde, light/dark brown, or black), and skin colour (fair, medium, or olive) on the left buttock were evaluated using a 12-point skin colour scale. The number of MN and freckles was counted using a standardised protocol from our previous studies [28,29]. The number of MN was investigated in 24 anatomical regions (except buttocks and external genitals) and categorised into groups based on sun exposure: maximal (face, neck, hands, ears, and shoulders), intermittent (chest, subscapular/lumbar regions, abdomen, outer upper arms, thighs, calves, and feet), and minimal (scalp, buttocks, armpits, inner forearms, palms, and soles). The skin examination was performed by three medical doctors. The validity of MN counting by medical doctors was ensured through training provided by an experienced dermatologist and co-author (S.V.). Clinical judgments regarding MN evaluation differed by less than 5% between the investigators.

All subjects were asked about their personal history of CM and whether their parents had pigmented moles. Sunbathing habits (time spent at the beach in summer), sunscreen use, sunlight sensitivity, tanning bed use (≥1 time), and the frequency of severe sunburns were assessed. The variable “number of sunburns” was documented based on self-reported history. It included all severe sunburn episodes (i.e., those resulting in painful erythema, as well as cases with skin peeling or blistering). The definition of “use of sunbeds” referred to self-reported use of a tanning bed one or more times. For the control group, all variables were assessed for the year prior to the study. For the case group, variables were assessed for the year preceding the confirmed diagnosis of CM. Based on the subjects’ responses regarding their skin’s reaction to sunlight, we determined their Fitzpatrick skin phototype (I–IV) [36]. For statistical analysis, these phototypes were combined into two categories (I–II and III–IV).

### 2.4. Statistical Analysis

Statistical data analysis was performed using IBM SPSS Statistics software (version 29.0.2.0). The Shapiro–Wilk test was used to assess the normality of quantitative variables. Student’s *t*-test for independent samples was used to compare normally distributed quantitative data in two groups, and data were summarised by presenting their mean with standard deviation (SD). Mann–Whitney U test was used to compare not normally distributed data, which were described by presenting their median (minimum value–maximum value). Categorical variables were compared using the Chi-square test (χ^2^) for homogeneity. Statistical significance was set at *p* < 0.05. A binary multivariate logistic regression model with backward elimination was used to determine statistically significant risk factors for the prognosis of CM. Model fit to data: the goodness-of-fit test (χ^2^ = 197.348, *p* < 0.001), Nagelkerke’s R^2^ = 0.618, and the Hosmer–Lemeshow test (χ^2^ = 11.577, *p* = 0.171) correctly classified 78.3% of CM cases and 87.5% of controls. Possible confounding variables such as age and sex were considered, as they may influence both sun exposure habits and CM risk. The case and control groups were homogeneous regarding these variables, and their inclusion in the regression model did not significantly affect the results; therefore, they were excluded from the final model.

## 3. Results

The sex distribution and mean age did not differ between the case and control groups (Table 1). Most participants were female, reflecting the proportion of females with CM in the Lithuanian health statistics [35]. Case subjects had a higher number of MN in both size categories, with a significantly higher median of MN > 2 mm, particularly in intermittently or maximally sun-exposed areas. Similarly, ≥30 freckles on the face, shoulders, and dorsal hands, as well as fair skin, were more common in the case group. Regarding sun exposure habits, tanning bed use, not using sunscreen, spending ≥2 h per day at the beach during vacations, and a higher median number of sunburns were all more frequent in cases (Table 1). Other variables, including age, sex, hair, and eye colour, Fitzpatrick skin type, and family history of numerous pigmented nevi, were not statistically significant.

**Figure 1:**
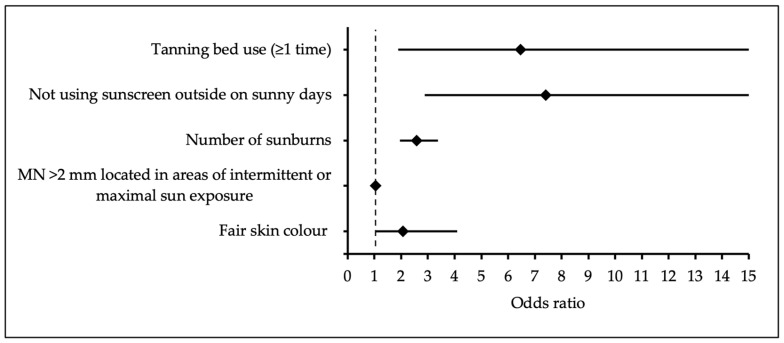
Forest plot graph of multivariate analysis of cutaneous melanoma predictors.

The multivariate binary logistic regression model identified five risk factors that statistically significantly increased the odds of CM: tanning bed use compared to non-use, lack of sunscreen use compared to regular use (*p* < 0.001), a higher amount of sunburn (*p* < 0.001), a greater number of MN > 2 mm in sun-exposed areas (*p* < 0.001), and fair skin compared with medium or olive skin (*p* = 0.004) (Table 2). A forest plot illustrating the predictors of CM is presented in Figure 1.

## 4. Discussion

UV radiation is strongly linked to the development of CM. It damages DNA through the formation of photoproducts and reactive oxygen species (ROS) and creates an immunosuppressive environment (e.g., antigen presentation impairment, the promotion of immunosuppressive cytokines), ultimately leading to mutagenic lesions [37,38]. UVA (315–400 nm), by penetrating deep into the dermis, primarily induces ROS. UVB (280–315 nm), with shorter wavelengths, forms photoproducts and is thought to be the main driver of melanomagenesis [39]. Although naturally present in sunlight, UVA and UVB radiation are also emitted by tanning beds.

One of the most significant findings in our study was the strong association between tanning bed use and CM risk. Comparable findings have been reported previously. For example, a prospective cohort study in Norway found that CM risk for females increased by 1.32-fold with a cumulative number of tanning sessions. Additionally, tanning bed use before age 30 raised CM risk by 1.31 times [20]. Ghiasvand et al. noted that both indoor tanning and lifetime number of sunbathing vacations were specifically linked to the trunk and lower limb CM risk increase [9]. Similarly, a case–control study in Poland reported a 2.65-fold higher CM risk for tanning bed use at an early age, which increased to 8.42-fold with prolonged solarium use over the years [14]. Moreover, retrospective data from the USA has shown that individuals with CM use tanning beds at least once, approximately twice as often as controls [7]. The dangers of sunbed use are also noted by Nurla et al., who report a female patient who developed CM after 12 months of intensive sunbed use (4–5 times per week) with tanning enhancers [40]. The authors emphasise the need for stricter sunbed use regulations, such as limits on UVA output, shorter exposure times, and stronger social media campaigns about the dangers of sunbed use.

Severe sunburns were also strongly linked to CM risk in our study, with each additional sunburn episode increasing risk by 2.57-fold. Sunburns have been described as a CM risk factor by various authors. Stenehjem et al. reported a significant dose–response relationship between sunburn frequency and increased risks of CM in Norwegian offshore workers [15]. Other studies conducted in Norway found that individuals with persistently high sunburn rates have a 1.50-fold increased risk of CM [16] and that the mean lifetime number of sunburns is associated with an increased risk of trunk and limb CM [9]. In France, Savoye et al. reported that experiencing six or more severe sunburns before age 25 is linked to a 2.7-fold increased CM risk [18]. Moreover, a prospective study in the USA reported a 2.41-fold increased CM risk in men with a history of severe sunburn, particularly on the trunk [17].

In our study, similar to other research, improper sunscreen application was associated with a 7.41-fold increase in CM risk. Ghiasvand et al. found that using sunscreen with a sun protection factor (SPF) ≥ 15 reduced CM risk by 0.67 times compared to SPF < 15. Moreover, the authors estimate that using SPF ≥ 15 sunscreen for 10 years could reduce CM incidence by 18% in women aged 40–75 [41]. Savoye et al. demonstrated that sunscreen use after age 25 was linked to a diminished protective effect and increased risks across multiple skin cancer types, including a 1.80-fold risk increase for CM [18].

Although not analysed in our study, occupational UV exposure (i.e., pilots exposed to cosmic and solar UVA radiation) [21] and ambient UV levels during lifetime [22] were also found to correlate with increased CM risk. Nonetheless, some studies have contradictory results when evaluating UV-related factors and CM risk. For example, prospective cohort studies in the USA did not observe a significant interaction between tanning bed use [19], cumulative UV exposure in childhood [26] and adulthood [19,25,26], and CM risk. Moreover, case–control studies from Australia and Europe suggest that occupational sun exposure does not increase the risk of CM [23,24].

Our study found that individuals with fair skin had a 2.06-fold higher CM risk compared to those with medium or olive skin, consistent with findings from previous studies [9,10,12]. The findings regarding the number of MN and their relationships with CM risk were consistent with our previously published results of case–control studies [30,31] and align closely with the findings reported by other authors [9,10,11,12,13]. However, the varying methodologies employed complicate direct comparisons. In our study, each additional MN >2 mm in sun-exposed areas increased CM risk by 1.05 times. Wei et al. analysed data from three prospective cohorts, where participants reported the number of MN on their left arm (>3 mm), both forearms (>3 mm), and lower legs (no size specification) [13]. The meta-analysis of combined data revealed an increase in CM risk with ≥1 MN in a dose-dependent trend—that is, the risk of CM increased progressively with the number of MN. The greatest CM risk was detected in individuals with ≥15 MN on the extremities, who had a 2.79-fold increased risk, compared to those without any extremity melanocytic nevi [9]. Olsen et al. found a 4.91-fold increased risk of invasive CM in individuals with a high self-reported melanocytic nevi density [10]. Hübner et al. documented a 1.3-fold increased likelihood of CM detection in those with ≥40 MN (≥2 mm diameter) [11].

The association of tanning bed use, sunburn occurrence, and risk of CM suggests that dermatologists, primary care physicians, and other healthcare professionals involved in related fields should take careful histories of UV exposure during routine assessments. A very important aspect is education, especially for young people, regarding the damage caused by UV radiation. There is a necessity to strengthen patient education regarding UV risks, have stricter control of tanning devices, and use targeted public health messaging. Moreover, current policies do not often clearly restrict the frequency of tanning sessions and lack enforcement against underage use, showing the urgent need for stricter supervision. Considering all of this, we can conclude that the results of our study are significant for target prevention programmes of CM.

Our study has several limitations. We assessed the sun exposure habits of the subjects retrospectively by means of a survey. Thus, we were unable to assess the cumulative effect of tanning bed exposure and the protective effect of different sunscreen protection factors (SPF) against CM. Furthermore, UV exposure was assessed during a one-year period for both subjects and controls. Therefore, it may not accurately reflect lifetime or childhood UV exposure. Another limitation of our study is that we did not assess the associations between CM risk and familial history, lifestyle factors such as diet, and smoking. However, despite these limitations, we have important results, published for the first time in relation to the Baltic region, that intermittent sun exposure and tanning bed exposure are important for the prevention of CM.

## 5. Conclusions

To our knowledge, we conducted the first case–control study in the Baltic countries that identified CM risk factors, including tanning bed use, lack of sunscreen, severe sunburns, and MN in sun-exposed areas. Our results highlight the need for targeted prevention strategies, such as stricter control of tanning devices, greater use of public health messaging on the harmful effects of UV radiation, and the implementation of more thorough skin examinations for high-risk individuals, such as those with a history of extensive tanning. Additionally, due to similarities of fair phenotypic traits, our results may also be applicable to other Baltic countries. Future case–control studies in Baltic countries are needed to investigate the cumulative effects of sun exposure.

## Figures and Tables

**Table 1:** Sun exposure habits and other risk factors in cutaneous melanoma patients and control subjects.

Characteristics	Control Group	Case Group	*p*-Value
	N = 182	N = 180	
Sex, % (n)			
Male	37.9 (69)	37.2 (67)	0.892
Female	62.1 (113)	62.8 (113)	
Total	182	180	
Age (years), mean (SD)			
Male	57.97 (8.20)	60.70 (13.50)	0.155
Female	57.34 (7.83)	58.88 (11.89)	0.249
Total	57.58 (7.95)	59.56 (12.51)	0.072 ^a^
Number of melanocytic nevi, median (range)			
Diameter 2–5 mm	4 (0–131)	19.5 (0–194)	<0.001 ^b^
Diameter ≥ 5 mm	0 (0–25)	3 (0–51)	<0.001 ^b^
Number of MN > 2 mm across varying sun-exposure areas, median (range)			
Minimal	0 (0–40)	1 (0–28)	<0.001 ^b^
Intermittent or maximal	5 (0–135)	23 (0–188)	
Freckles on face, shoulders, and dorsal surface of the hands, % (n)			
<30	64.3 (117)	42.8 (77)	<0.001
≥30	35.7 (65)	57.2 (103)	
Skin colour, % (n)			
Fair	30.2 (54)	48.6 (87)	<0.001
Medium/olive	69.8 (125)	51.4 (92)	
Hair colour, % (n)			
Blond/red	10.1 (18)	16.8 (30)	0.069
Light brown	41.9 (75)	45.3 (81)	
Dark brown/black	48.0 (86)	38.0 (68)	
Eye colour, % (n)			
Blue/grey	66.7 (118)	65.9 (118)	0.863
Green	19.2 (34)	21.2 (38)	
Brown	14.1 (25)	12.8 (23)	
Skin type (Fitzpatrick scale), % (n)			
Type I/Type II	44.7 (80)	41.3 (74)	0.552
Type III/Type IV	55.3 (99)	58.7 (105)	
Biological parent with ≥50 pigmented moles, % (n)			
At least one	31.7 (40)	32.9 (46)	0.847
Neither	68.3 (86)	67.1 (94)	
Tanning bed use (≥1 time), % (n)			
No	95.6 (174)	82.8 (149)	<0.001
Yes	4.4 (8)	17.2 (31)	
Not using sunscreen outside on sunny days, % (n)			
Yes	66.7 (118)	88.1 (156)	<0.001
No	33.3 (59)	11.9 (21)	
Time spent daily at the beach while vacationing, % (n)			
<2 h	41.4 (70)	23.9 (38)	<0.001
≥2 h	58.6 (99)	76.1 (121)	
Number of sunburns, median (range)	0 (0–6)	2 (0–6)	<0.001 ^b^

^a^ Student’s *t*-test for independent samples; ^b^ Mann–Whitney U test; otherwise, Chi-square test (χ^2^) for homogeneity; n—the total number of individuals or observations in the sample; SD—standard deviation; mm—millimetres in diameter.

**Table 2:** Multivariable analysis of cutaneous melanoma predictors.

Variable	B	Odds Ratio	95% Confidence Interval		*p*-Value
			Lower Bound	Upper Bound	
Tanning bed use (≥1 time)	1.87	6.46	1.89		0.004
			22.96		
Not using sunscreen outside on sunny days	2.003	7.41	2.88		<0.001
			19.09		
Number of sunburns	0.946	2.57	1.96		<0.001
			3.38		
MN >2 mm located in areas of intermittent or maximal sun exposure	0.051	1.05	1.03		<0.001
			1.07		
Fair skin colour	0.722	2.06	1.03		0.040
			4.09		

B—coefficient of the model; OR—odds ratio; CI—confidence interval of OR.

## Data Availability

The data presented in this study are available on request from the corresponding author.

## Abbreviations

The following abbreviations are used in this manuscript:

AMN	Atypical Melanocytic Nevi
CI	Confidence Interval
CM	Cutaneous Melanoma
DNA	Deoxyribonucleic Acid
IBM SPSS	International Business Machines Statistical Package for the Social Sciences
LSMU	Lithuanian University of Health Sciences
LSMUL KK	Lithuanian University of Health Sciences Kauno Klinikos (Hospital of LSMU)
MN	Melanocytic Nevi
OR	Odds Ratio
ROS	Reactive Oxygen Species
SD	Standard Deviation
SPF	Sun Protection Factor
UV	Ultraviolet
χ^2^	Chi-Square
